# IL-8-induced CXCR2 down-regulation in circulating monocytes in hepatocellular carcinoma is partially dependent on MAGL

**DOI:** 10.1186/s12885-023-11109-5

**Published:** 2023-07-04

**Authors:** Chong Zhong Liu, Xing Bao Liu, Jun Sun, Chao Qun Yu, Jing Chun Yao, Zhong Liu, Jing Cheng Hao

**Affiliations:** 1 Department of Hepatobiliary Surgery, The Second Hospital, Cheeloo College of Medicine, Shandong University, 247 Beiyuan Street, Jinan, Shandong 250033 PR China; 2 Department of Hepatobiliary Surgery, Feixian People’s Hospital, 71 Jiankang Road, Feixian, 273499 PR China; 3 State Key Laboratory of Generic Manufacture Technology of Chinese Traditional Medicine, Lunan Pharmaceutical Group Co. Ltd., 209 Hongqi Road, Linyi, 276006 PR China; 4 Key Laboratory of Colloid and Interface Chemistry, Ministry of Education, Shandong University, Jinan, 250100 PR China

**Keywords:** Primary hepatic carcinoma, Monocytes, CXCR2, IL-8, Monoacylglycerol lipase

## Abstract

**Background:**

CXC-chemokine receptor 2 (CXCR2) expression was found to be down-regulated on circulating monocytes of cancer patients. Here, we analyze the percentage of CD14^+^CXCR2^+^ monocyte subsets in hepatocellular carcinoma (HCC) patients, and investigate the mechanisms that regulate CXCR2 surface expression on monocytes and its biological function.

**Methods:**

Flow cytometry was used to analyze the proportion of the CD14^+^CXCR2^+^ subset from the total circulating monocytes of HCC patients. Interleukin 8 (IL-8) levels were measured from serum and ascites, and their correlation with the CD14^+^CXCR2^+^ monocyte subset proportion was calculated. THP-1 cells were cultured in vitro and treated with recombinant human IL-8 and CXCR2 surface expression was analyzed. CXCR2 was knocked down to examine how it affects the antitumor activity of monocytes. Finally, a monoacylglycerol lipase (MAGL) inhibitor was added to analyze its effect on CXCR2 expression.

**Results:**

A decrease in the proportion of the CD14^+^CXCR2^+^ monocyte subset was observed in HCC patients compared with healthy controls. CXCR2^+^ monocyte subset proportion was associated with the AFP value, TNM stage, and liver function. Overexpression of IL-8 was observed in the serum and ascites of HCC patients, and negatively correlated with CXCR2^+^ monocyte proportion. IL-8 decreased CXCR2 expression in THP-1 cells, contributing to decreased antitumor activity toward HCC cells. MAGL expression in THP-1 cells was up-regulated after IL-8 treatment, and the MAGL inhibitor partially reversed the effects of IL-8 on CXCR2 expression.

**Conclusions:**

Overexpression of IL-8 drives CXCR2 down-regulation on circulating monocytes of HCC patients, which could be partially reversed by a MAGL inhibitor.

## Background

Hepatocellular carcinoma (HCC) is a digestive system malignancy with high morbidity and mortality rates, ranking 7^th^ and 3^rd^, respectively, among the world's most common malignant tumors in 2018 [1]. Additionally, it is one of the four most common malignant tumors in China. The tumor microenvironment (TME) generally shows immunosuppressive activity in HCC, leading to immune escape and resistance to immune-based treatments. As one of the important component in TME, tumor associated macrophages (TAMs) contribute to weakened anti-tumor immune responses through the crosstalk with neighboring cells [2]. In short, TAMs inhibit the tumor killing activity of T cells and NK cells and promote the recruitment of Treg cells [3, 4]. In addition, TAMs promote the stemness and epithelial-mesenchymal transition (EMT) of tumor cells, leading to a more aggressive phenotype [5]. Most TAMs in HCC originate from circulating monocytes which are recruited by chemokines [6]. Several recent studies have demonstrated that modulating peripheral blood monocyte phenotype and function can affect the composition of TAMs subsets and antitumor activity [7, 8]. Therefore, circulating monocytes could be a potential target for macrophage-targeted antitumor immunotherapy.

IL-8 is overexpressed in both the tumor microenvironment and peripheral circulation of HCC patients and is associated with poor prognosis [9]. Overexpression of IL-8 promoted the epithelial-mesenchymal transition and the stem cell-like properties of HCC cells, and promoted the distant metastasis of HCC [10]. The major receptors of IL-8, CXCR1 and CXCR2 were also overexpressed in HCC cells and were involved in the invasive and migratory behavior of HCC [11]. In addition to tumor cells, CXCR1 and CXCR2 are also important chemokine receptors of myeloid immune cells, such as neutrophils and monocytes. The imbalance composition and abnormal function of immune cells infiltrating local tumors caused by abnormal expression of chemokine receptors were one of the key factors leading to tumor immune escape [12, 13]. However, the expression of CXCRs on immune cells of HCC patients has not been well studied.

In this study, we examined the proportion of a specific CD14^+^CXCR2^+^ monocyte subset in the peripheral blood and ascites of HCC patients, detected their correlation with IL-8 levels, and explored the relationship between this special cell subset with clinicopathological characteristics of HCC patients. Additionally, we verified the possible regulators of monocyte CXCR2 expression via in vitro experiments.

## Methods

### Patients and sample collection

Forty patients with HCC hospitalized in our hospital from January to December 2021 were enlisted, with an average age of 57.2 ± 7.7. All of them were primarily-diagnosed cases based on postoperative pathological examinations, and their tumors were staged according to the AJCC Cancer Staging Manual (8^th^ edition) [14]. No patients previously received associated treatments. Patients with other acute or chronic diseases were excluded. EDTA-2 K anticoagulated venous peripheral blood was drawn from patients before the operation. Anticoagulated peripheral blood was also drawn from 23 cases of age-matched healthy volunteers as healthy control group. Six out of 40 HCC patients were selected for ascites collection.

### Materials

The MAGL inhibitor MAGL11 is synthesized in Lunan Pharmaceutical Group Co. Ltd. The IC_50_ of MAGL11 to inhibit MAGL is 9.735 nM, as determined by Monoacylglycerol Lipase Inhibitor Screening Assay Kit (Cayman Chemical). 2-Arachidonylglycerol (2-AG) was purchased from Tocris. Anti-human CD14 (Clone: MΦP9)-PE fluorescent mAb was purchased from Becton Dickinson. Anti-human CXCR2 (Clone: 5E8/CXCR2)-APC fluorescent mAb was purchased from Biolegend.

### Flow cytometry

100μL anticoagulated blood was labeled with CD14-PE and CXCR2-APC monoclonal antibodies, incubated at room temperature in the dark for 20 min, and then lysed with red blood cells. After washing and resuspension, the expression levels of CD14^+^CXCR2^+^ cell subsets in the entire CD14^+^ monocyte population were detected by flow cytometry, and data analysis was performed using FlowJo software.

### Enzyme-linked immunosorbent assay

Serum of patients with HCC and healthy controls, or ascites of patients with HCC, were collected and centrifuged at 2,000 × g for 10 min, and the supernatant was harvest. The IL-8 concentration was detected using a commercial ELISA kit of human IL-8 (Elabscience, China), according to the manufacturer’s instructions.

### Cell culture

Cell lines of THP-1 human monocytes and HepG2 human hepatocellular carcinoma were purchased from Chinese Biological and Medical Cell Resource Center (Shanghai, China). The cells were cultured in RPMI-1640 medium containing 10% FBS at 37 °C with 5% CO_2_ and passaged every 2–3 days. In some experiments, MAGL inhibitor MAGL11 (25 nM) and/or 2-AG (100 uM) was added to THP-1 cells 1 h before recombinant human IL-8 (rhIL-8, 20 ng/mL) application. THP-1 cells were collected after 24 h of treatment.

### CXCR2-knockdown

The CXCR2-knockdown lentiviral plasmid was constructed by the Genechem Company (Shanghai, China) and transfected into THP-1 cell line with a MOI of 50. The stably transfected cell lines were screened with puromycin. After two weeks, the transfection efficiency exceeded 95%, as verified by flow cytometry, and the positive rate of CXCR2 in THP-1 cells after transfection was less than 10%.

### Apoptosis assay

HepG2 cells were placed in a 6-well plate at a density of 2 × 10^5^/well and incubated for 24 h in a 37 °C incubator with 5% CO_2_. After the adherent cells growth, the medium was moved, washed twice by PBS, and then 2 × 10^6^/well control of CXCR2-knockdown THP-1 cells, suspended in 2 mL of complete RPMI-1640 medium, were added into the same well. In some groups, control of CXCR2-knockdown THP-1 cells were treated with recombinant human IL-8 (20 ng/mL) for 24 h and then co-cultured with HepG2 cells. After 16 h, the adherent HepG2 cells were digested with trypsin and stained with Annexin V and PI double staining kit. The level of apoptosis was detected by flow cytometry.

### RT-PCR

RNA was extracted with RNAfast200 total RNA extraction kit (Fastagen, China) and reverse transcribed to synthesize cDNA. Real-time quantitative PCR (RTq-PCR) was performed on a CFX96 fluorescence PCR instrument (Bio-Rad). The internal reference was GAPDH. The primer sequences were shown below: MAGL, forward: 5′-TCT GAC TTC CAC GTT TTC GTC-3′, reverse 5′-AGA ACC AGA GGC GAA ATG AGT-3′; GAPDH, forward: 5’-TCG GAG TCA ACG GAT TTG GTC GTA-3’, reverse: 5’-CTT CCT GAG TAC TGG TGT CAG GTA-3’.

### Statistical analysis

Statistical analysis was performed using SPSS17.0 and GraphPad Prism 8.0 software. Normally distributed measurement data are expressed as‾X ± SD and were analyzed using a *t*-test or one-way ANOVA. The correlation between the CD14^+^CXCR2^+^ cells proportion and IL-8 concentration was analyzed using the Pearson correlation coefficient. In addition, the differences in clinicopathological characteristics of the CD14^+^CXCR2^+^ high and low groups were analyzed using an *X*^*2*^ test or Fisher's exact test. *p* < 0.05 was considered statistically significant.

## Results

### Proportion of CD14^+^CXCR2^+^ monocyte subsets in the peripheral blood of patients and healthy controls

**Fig. 1:**
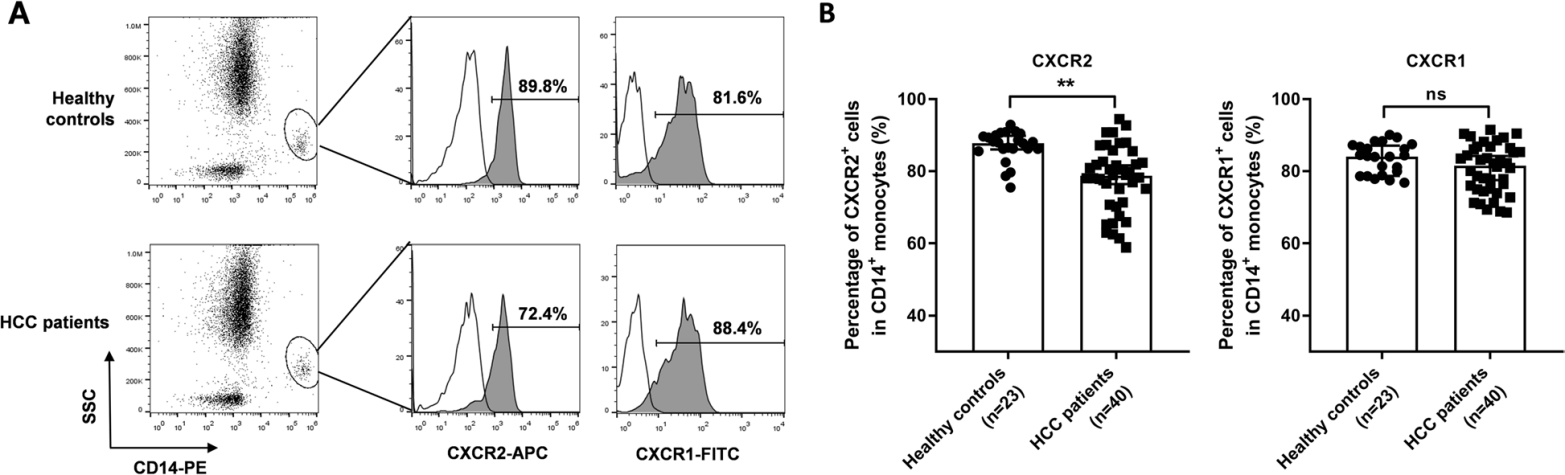
CXCR2^+^ cell subsets proportion in CD14^+^ peripheral monocytes of HCC patients and healthy controls. **A** Typical images of CXCR2^+^ cells in peripheral CD14^+^ monocytes of healthy controls and HCC patients; **B** Quantitative statistics of the proportion of CXCR2^+^ cells in peripheral CD14^+^ monocytes of healthy controls and HCC patients. ^**^, *p* < 0.01

The CXCR2^+^ cell subset in the peripheral blood of healthy controls accounted for (86.75 ± 4.27)% of all CD14^+^ peripheral blood monocytes, while the proportion in patients with HCC was (77.77 ± 9.17)% (Fig. 1A), which was significantly lower than that of the healthy control group (*p* < 0.01, Fig. 1B). Compared with CXCR2, the positive percentage of CXCR1, another important receptor of IL-8, did not show difference on CD14^+^ peripheral monocytes between healthy controls and HCC patients (Fig. 1A and B).

### Correlation between the percentage of CXCR2^+^ monocytes in peripheral blood and clinicopathological features of HCC

Taking the median percentage of CXCR2^+^ monocytes (78.76%) as the cutoff value, the patients with HCC were divided into two groups (CXCR2^+^ low and CXCR2^+^ high), and the differences in clinicopathological characteristics of the two groups were compared. As shown in Table 1, the AFP level, liver function, and TNM stage in the CXCR2^+^ low group were significantly different from those in the CXCR2^+^ high group, while other clinicopathological features such as age, gender, tumor number, tumor diameter, and HBsAg were not significantly different.

**Table 1:** Correlation analysis of CXCR2^+^ peripheral monocytes and clinicopathological characteristics of HCC patients

	**CXCR2**^**+**^** low**	**CXCR2**^**+**^** high**	***p* value**
Age			
< 60	10	12	0.525
≥ 60	10	8	
Gender			
Male	17	16	0.677
Female	3	4	
HBsAg			
(-)	3	4	0.225
( +)	17	16	
AFP (μg/L)			
< 400	12	20	0.002
≥ 400	8	0	
Tumors number			
1	13	18	0.064
> 1	7	2	
Tumor diameter (cm)			
≤ 3	4	6	0.150
3 ~ 5	5	9	
> 5	11	5	
Liver function			
Child A	10	18	0.016
Child B	6	2	
Child C	4	0	
TNM staging			
I (Ia ~ Ib)	3	12	0.001
II (IIa ~ IIb)	8	8	
III (IIIa ~ IIIb)	9	0	

### Correlation of serum and ascites serum IL-8 levels with the percentage of CXCR2^+^ monocytes in patients with HCC

**Fig. 2:**
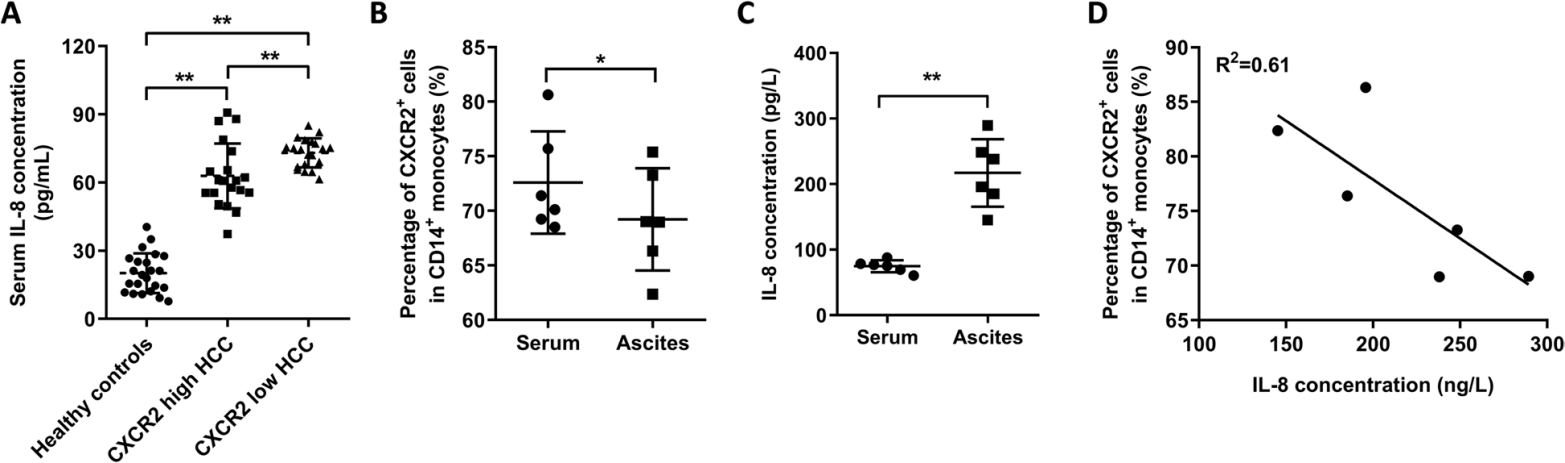
Correlation of serum and ascites IL-8 levels with CXCR2^+^ monocytes proportion in HCC patients. **A** Serum IL-8 expression levels in healthy controls, CXCR2-low and CXCR2-high HCC patients. ^*^, *p* < 0.05, ^**^, *p* < 0.01. **B** Percentage of CXCR2^+^ monocytes in peripheral blood and ascites of HCC patients. ^*^, *p* < 0.05. **C** Serum and ascites IL-8 levels of HCC patients. ^**^, *p* < 0.01. **D** Correlation analysis between the proportion of CXCR2^+^ monocytes and IL-8 levels in ascites of HCC patients

Our results showed that serum IL-8 levels in patients with HCC were significantly higher than those in healthy controls, regardless of whether proportions of CXCR2^+^ monocytes were low or high. In comparison, serum IL-8 levels in patients in CXCR2^+^ low group were significantly higher than those in patients in CXCR2^+^ high group (Fig. 2A). The percentage of CXCR2^+^ monocytes in the patient's ascites was lower than that in the peripheral blood (Fig. 2B). IL-8 levels in ascites of HCC patients were significantly higher than those in their serum (Fig. 2C), while IL-8 level in ascites was negatively correlated with the proportion of CXCR2^+^ monocytes (*R*^*2*^ = 0.61, Fig. 2D).

### IL-8 inhibits the surface expression of CXCR2 in monocytes

**Fig. 3:**
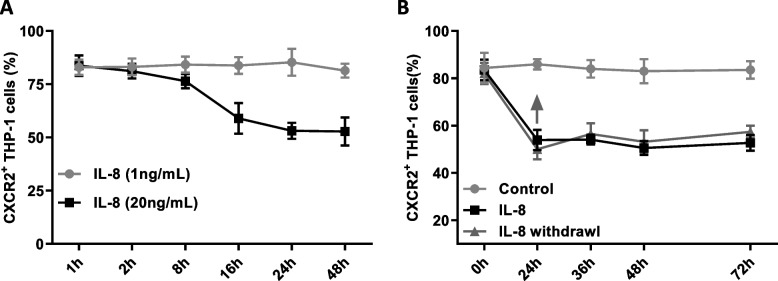
The effect of exogenous human recombinant IL-8 on the expression of CXCR2 in THP-1 cells. **A** The positive percentage of CXCR2 on THP-1 cells, treated with 1 ng/mL or 20 ng/mL IL-8 at different times, as detected by flow cytometry. **B** The effect of IL-8 withdrawal on the expression of CXCR2 on the surface of THP-1. Control: THP-1 without any treatment; IL-8: 20 ng/mL human recombinant IL-8 acts on THP-1 cells; IL-8 withdrawal: 20 ng/mL human recombinant IL-8 acted on THP-1 cells for 24 h, after which the medium was changed, and continued for a corresponding time. The upward arrow represented the time point of IL-8 withdrawal

The THP-1 monocyte cell line was cultured in vitro and stimulated with different concentrations of exogenous IL-8. The results showed that 1 ng/mL IL-8 did not affect the expression of CXCR2 in THP-1 cells, while 20 ng/mL IL-8 treatment for 8 h significantly down-regulated the expression of CXCR2 in THP-1 cells, reaching a steady state after 24 h of treatment (Fig. 3A). When the THP-1 cells were treated with 20 ng/mL IL-8 for 24 h and the exogenous IL-8 was withdrawn, the inhibitory state of CXCR2 expression by IL-8 could be maintained for at least 48 h (Fig. 3B).

### Down-regulation of CXCR2 expression inhibited the killing activity of monocytes on hepatocellular carcinoma cells

**Fig. 4:**
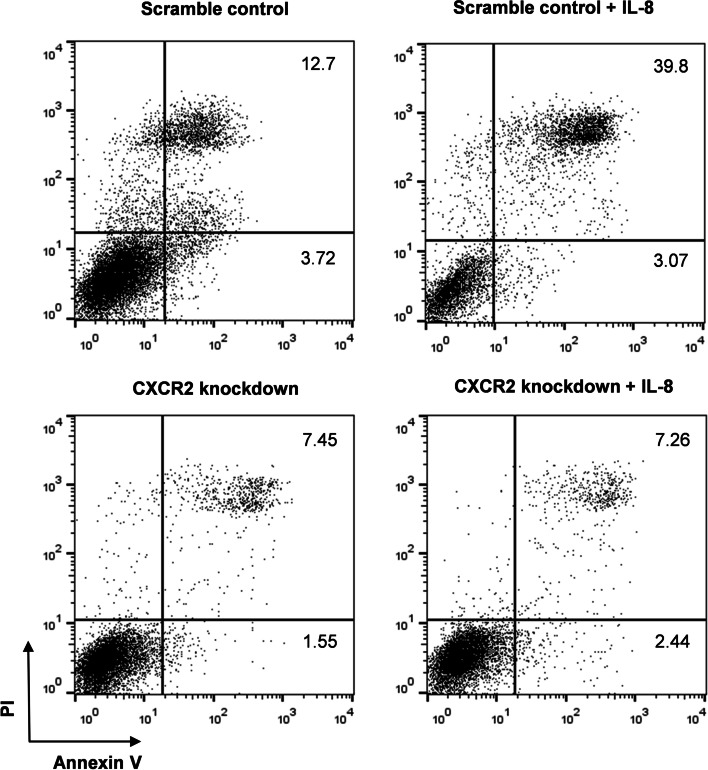
Killing activity of CXCR2 knockdown THP-1 cells on HCC. Typical apoptosis images of HepG2 cells co-cultured with scramble control or CXCR2-knockdown THP-1 cells with/without IL-8 (20 ng/mL) treatment. Scramble control: HepG2 cells co-cultured with THP-1 cells transfected into control plasmid; CXCR2 knockdown: HepG2 cells co-cultured with THP-1 cells transfected into CXCR2 knockdown plasmid

The CXCR2-knockdown or control THP-1 cells were treated with IL-8 and then co-cultured with the primary hepatoma cell line HepG2. After 16 h, apoptosis analysis showed that co-culturing with IL-8 pre-treated control THP-1 cells enhanced the apoptosis level of HepG2 cells, compared with those without IL-8 treatment. The apoptosis percentage of HepG2 cells co-cultured with CXCR2-knockdown THP-1 cells was lower than those co-cultured with control THP-1 cells. Moreover, pre-treatment with IL-8 did not increase the apoptosis of HepG2 cells co-cultured with CXCR2-knockdown THP-1 cells (Fig. 4).

### MAGL inhibitor reversed the down-regulation of CXCR2 expression in monocytes by IL-8

**Fig. 5:**
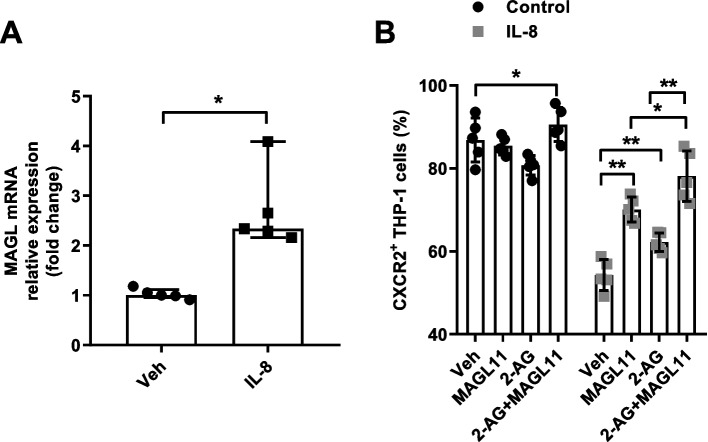
MAGL inhibitor reverses the down-regulation of CXCR2 in monocytes by IL-8. **A** MAGL mRNA expression in IL-8-treated THP-1 cells and control THP-1 cells. **B** Effects of MAGL inhibitor MAGL11 and/or 2-AG on CXCR2 expression in IL-8-treated and non-IL-8-treated THP-1 cells. ^*^, *p* < 0.05. ^**^, *p* < 0.01

In monocytes/macrophages, the endocannabinoid-degrading enzyme MAGL is significantly up-regulated under the stimulation of inflammation signals, and plays an important role in processes such as liver fibrosis and tissue damage repair [15]. MAGL expression was significantly up-regulated in THP-1 cells treated by exogenous IL-8 (Fig. 5A). The addition of a MAGL inhibitor (MAGL11) or 2-AG, the main substrate of MAGL, did not significantly affect CXCR2 expression on THP-1 cells. However, CXCR2 expression was up-regulated under the collaborative treatment of MAGL11 and 2-AG. Both MAGL11 and 2-AG significantly reversed the down-regulation of CXCR2 expression by IL-8, and the combined application showed stronger effect than single factor (Fig. 5B).

## Discussion

Although circulating monocytes are the primary source of tumor-associated macrophages [13], little is known about the factors that regulate their phenotype and function. In this study, a decreased percentage of peripheral blood CD14^+^CXCR2^+^ monocytes in patients with HCC was observed. This percentage was correlated with the AFP levels, liver function, and TNM stage of patients. IL-8 levels were significantly up-regulated in patients with HCC and were negatively correlated with the percentage of CD14^+^CXCR2^+^ monocytes in the peripheral blood or ascites. In vitro cell experiments showed that IL-8 was one of the key factors inhibiting the expression of CXCR2 in monocytes. The down-regulation of CXCR2 expression affected the tumor-killing activity of monocytes on primary hepatoma cells. The involvement of CXCR2 in the regulation of biological functions of circulating monocytes could provide target and evidence in monocyte/macrophage-based antitumor immunotherapies.

The expression of chemokine receptors was markedly altered and had important pathophysiological implications in various diseases, such as infections, tumors, and autoimmune diseases [16]. In this study, the percentage of CD14^+^CXCR2^+^ monocytes in peripheral blood was significantly downregulated in patients with HCC compared with that in healthy controls. CXCR2 is a major chemokine receptor for neutrophils to mediate their migration [17], but it has not been well studied on monocytes. Our results demonstrate that there was a high level of CXCR2 expression on the surface of peripheral blood monocytes, which suggests its role in the migration of monocytes. In patients with traumatic brain injury (TBI), CXCR2 expression on circulating monocytes was transient up-regulated after surgery, and was involved with increased chemotaxis toward cerebrospinal fluid (CSF) and secretion of pro-inflammatory cytokines [18]. This suggests that CXCR2 can affect cytokine secretion of monocytes in addition to their chemotaxis. The change of cytokine secretion profile may be one of the mechanisms that CXCR2 knockdown led to decreased tumor killing activity. In addition, we detected monocytes in ascites of patients with HCC and found that their CXCR2 expression level was lower than that of circulating monocytes. In a recent study involving immune cells in ascites and peripheral blood of patients with HCC, it was found that the majority of monocyte-macrophages in ascites came from tumor tissue rather than peripheral blood through the landscape and dynamic analysis at the single cell level [19]. Therefore, analyzing monocytes in ascites may better reflect their characteristics in TAMs. This indicates that CXCR2 down-regulation in monocytes could be a common phenomenon occurs in many types of tumors and regulates macrophage-mediated antitumor immune activity. In future studies, we will further test this hypothesis in other tumors and continue to explore the possible roles of monocyte/macrophage-derived CXCR2 expression in tumorigenesis and development.

IL-8 is the major ligand of CXCR2 and is highly expressed in various types of tumors [20]. Overexpression of IL-8 was found in Hepatitis B-associated hepatocellular carcinoma and led to enhanced endothelial permeability to facilitate tumor vascular invasion [21]. In this study, serum IL-8 levels in patients with primary liver cancer were significantly higher than those in healthy controls, and its level was higher in patients with low CXCR2^+^ monocytes percentage. In ascites of HCC patients, IL-8 levels were significantly higher than that in peripheral blood and were negatively correlated with the proportion of CD14^+^CXCR2^+^ monocytes. These results demonstrate that IL-8 could be one of the factors decreasing the expression of CXCR2 in monocytes, and our in vitro experiments confirmed this hypothesis. It should be noted that IL-8-treated monocytes induced an increase in apoptosis of HepG2, as shown in our study. A recent study has confirmed that IL-8 promotes pro-inflammatory activity of human monocytes/macrophages, including inhibiting the expression of CD16 and CD124 (IL-4RA), and significantly promoting the expression of IFN-g receptor CD119 and the secretion of pro-inflammatory cytokines [22], and therefore may explain how IL-8 promotes the cytotoxicity of THP-1 cells. Importantly, we observed an interesting phenomenon that IL-8 treatment only promoted the apoptosis of HepG2 cells co-cultured with THP-1 cells of the scramble control group, which express high level of CXCR2 and simulate circulating monocytes of healthy controls; while in the THP-1 cells with CXCR2 knock-down, which simulates the monocytes of HCC patients, the regulatory effect of IL-8 on the cytotoxicity towards HepG2 was lost.

MAGL is a key degrading enzyme of endocannabinoids, which can convert 2-arachidylglycerol to arachidonic acid, regulating prostaglandin metabolism. MAGL is expressed in macrophages. The specific knockout of MAGL in macrophages or the application of MAGL inhibitors suppressed macrophage pro-inflammatory cytokine expression and inhibited the macrophage-mediated liver fibrosis process [23], suggesting that targeting MAGL attenuates the macrophage-mediated local inflammatory state. Our findings show that IL-8 can promote the expression of MAGL in THP-1. MAGL inhibitors reversed the down-regulation of CXCR2 in THP-1 cells caused by exogenous IL-8 but did not affect the expression of CXCR2 in THP-1 without IL-8, suggesting that MAGL could interfere with IL-8 mediated inflammatory signaling pathways. Therefore, MAGL may be an important target during IL-8-mediated immune escape in patients with HCC.

## Conclusion

In conclusion, we found that down-regulation of the proportion of CD14^+^CXCR2^+^ monocytes in patients with HCC was associated with the antitumor immune activity. Overexpressed IL-8 could be a key factor leading to the down-regulation of CXCR2 expression. This study provides evidence for the abnormal immune status and immune escape mechanism of HCC, and lays the groundwork for new immunotherapy strategies against tumors. In future research, we will further explore the comprehensive roles of monocyte-derived CXCR2 in the progression of HCC by utilizing in vitro cell experiments and animal models.

## Data Availability

The datasets used and/or analysed during the current study are available from the corresponding author on reasonable request.

## Abbreviations

HCC: Hepatocellular Carcinoma
IL-8: Interleukin 8
CXCR2: CXC-chemokine Receptor 2
APC: Antigen Presenting Cells
TME: Tumor Microenvironment
TAMs: Tumor Associated Macrophages
AFP: Alpha Fetoprotein
MAGL: Monoacylglycerol Lipase
